# Ultrastructure and Transcriptomic Analysis Reveal Alternative Pathways of Zona Radiata Formation in *Culter alburnus* with Different Spawning Habits

**DOI:** 10.3390/biology14080987

**Published:** 2025-08-03

**Authors:** Yan Zhao, Ge Xue, Yanghui Peng, Jia Zhang, Feng Chen, Yeke Wang, Jun He, Jun Chen, Ping Xie

**Affiliations:** 1 Donghu Experimental Station of Lake Ecosystems, State Key Laboratory of Freshwater Ecology and Biotechnology, Institute of Hydrobiology, Chinese Academy of Sciences, Wuhan 430072, China; zhaoyan202506@126.com (Y.Z.); xuege319@163.com (G.X.); pengyanghui@ihb.ac.cn (Y.P.); zhangjia001@ihb.ac.cn (J.Z.); chenfeng248@126.com (F.C.); yekewang@126.com (Y.W.); hejun@ihb.ac.cn (J.H.); 2 University of Chinese Academy of Sciences, Beijing 100049, China; 3 College of Life Sciences, Hebei University, Baoding 071002, China

**Keywords:** spawning habits, zona radiata, oogenesis, *Culter alburnus*

## Abstract

Spawning diversity plays an essential role in fish survival and reproduction, which contributes to the exceptional diversity of teleosts among vertebrates. Different zona radiata structures reflect the adaptability of fish to the environment of spawning and early embryonic development. The morphological and transcriptional characteristics of fish follicle development between different spawning habits, particularly the zona radiata variations, have been poorly documented. In this study, we integrated histology and transcriptomics to investigate the differences in the zona radiata structure and gene expression profiles among follicles from different spawning habits of *Culter alburnus*. Our results revealed that stage Ⅲ was the crucial period for zona radiata thickening and structure differentiation. Transcriptomic analyses of adhesive and semi-buoyant eggs at stage Ⅲ revealed a significant upregulation of genes involved in glycoprotein synthesis, extracellular matrix formation, and regulation of protease activity in adhesive eggs, such as the *wfdc* and *a2ml* gene family. This upregulation likely underpins the thicker zona radiata in adhesive eggs, facilitating their attachment to substrates. This study represents the first elucidation of the ultrastructure of the zona radiata and gene expression patterns in different developmental stages of adhesive and semi-buoyant eggs of *Culter alburnus*, offering new perspectives for aquaculture research in understanding fish reproductive adaptations.

## Simple Summary

Fishes exhibit diverse spawning habits, including adhesive, semi-buoyant, and demersal eggs. Key characteristics, such as egg stickiness and zona radiata thickness, reflect strong adaptation to different reproductive environments. The zona radiata is a non-cellular structure of fish eggs, serving to prevent polyspermy and protect the fertilized eggs. In this study, we selected *Culter alburnus*, a representative species with both adhesive and semi-buoyant egg-laying populations, to explore the molecular basis of egg-type differentiation. By integrating histological observations and comparative transcriptomic analyses, the results indicate that stage Ⅲ of oogenesis is critical for the formation of structural differences in the zona radiata. During this stage, genes related to glycosaminoglycan synthesis and protease inhibitors were significantly upregulated in adhesive eggs, suggesting their potential role in egg-type differentiation. These findings offer new insights into the regulatory mechanisms underlying egg-type differentiation, particularly the structural divergence of the zona radiata.

## 1. Introduction

Reproductive strategies, such as egg-spawning habits, reflect the adaptive responses of the Cyprinidae family to environmental factors [1,2,3]. Cyprinidae stands as the world’s largest family in terms of freshwater fish diversity [4], encompassing various spawning types, including semi-buoyant eggs, adhesive eggs, and demersal eggs [3,5]. Semi-buoyant eggs are typically spawned in river environments and require sufficient current velocity to keep in suspension [6], while adhesive eggs are spawned in lentic water bodies [3]. For East Asian cyprinids, the diversity in spawning habits represents an adaptive strategy within the complex potamo-lacustrine ecosystems of the middle and lower reaches of the Yangtze River basin [1,3]. Investigating mechanisms underlying the variation in egg types aids in comprehending mechanisms driving the evolution of biodiversity in the Yangtze River, particularly the radiation of fish species, under the influence of intensified monsoonal climates [2,7].

The zona radiata (ZR), also referred to as the egg envelope or chorion in fish, is an extracellular matrix comprising protein and glycoprotein layers [8]. Comparative studies have indicated that variations in ZR morphology and thickness may represent adaptations to diverse ecological conditions [9,10]. Adhesive eggs typically develop a thicker ZR, with specialized surface structures such as nodular protrusions or adhesive layers after fertilization [1,11,12,13]. The thicker ZR could help prevent prehatching damage from contact with hard structures in the environment [9,10]. In addition, glycoproteins and mucopolysaccharides (glycosaminoglycans (GAGs)) have been reported to contribute to the development of egg adhesiveness [1,11,14]. The difference in ZR structure between adhesive eggs and non-adhesive eggs is probably determined by two key factors: the deposition process of zona radiata proteins that occurs during oogenesis [15] and the biochemical modifications that take place during the fertilization process induced by a cortical reaction [16,17,18,19].

Research on ZR formation has primarily focused on its ultrastructure and major molecular constituents, the zona pellucida (ZP) proteins [10,20,21,22,23,24,25,26]. ZR generally comprises 2–4 layers, with different subtypes of ZP proteins contained within each layer [10,24,26]. At fertilization, ZR structural modifications are triggered, such as hardening and formation of an adhesive layer in some adhesive eggs, mediated by cortical alveoli contents released during the cortical reaction [11,12,19,27]. From fertilization to zygotic genome activation (ZGA), there is minimal transcriptional activity [28,29]. During this period, maternal factors, such as oocyte mRNA and proteins synthesized pre-fertilization, play an important role in modifying the ZR structure. Therefore, the analysis of differences in the transcriptional level for mechanisms underlying the ZR structure, focusing on both the initial substance deposition and post-fertilization modification, should be more appropriately conducted in oocytes or follicles. A comparative transcriptome across the different developmental stages of follicles has been employed to analyze the development and maturation of oocytes, addressing issues such as lipid droplet formation [30,31]. However, analyses of ZR formation-related genes during oogenesis have only been conducted within single egg-type species, indicating that *zp* genes are usually highly expressed at the early stage of follicle development [32,33,34].

*Culter alburnus*, a freshwater batch-spawning fish of the Cyprinidae family, exhibits asynchronous ovarian development, where oocytes at various maturation stages coexist, enabling multiple spawning events within a spawning season. Notably, *C. alburnus* has two ecotypes that differ in spawning habits. Individuals from the Liangzi Lake, Chao Lake, Taihu Lake, and Poyang Lake populations produce adhesive eggs (*C. alburnus* adhesive, Cala) that develop adhesiveness immediately and stick to aquatic plants or rocks, while those from the Xingkai Lake, Hanjiang River, and Yuanshui River populations produce semi-buoyant eggs (*C. alburnus* semi-buoyant, Calb) that hydrate and absorb water to disperse in floating rivers [35,36]. Current comparative transcriptomic analyses of ZR structure differences in *C. alburnus* with distinct egg types are limited to mature ovaries, lacking temporal expression profiles of relevant genes across follicular developmental stages [1]. In this study, we conducted a comparative analysis of the variances in ZR morphology and gene expression patterns between follicles of *C. alburnus* with different spawning habits at different developmental stages during oogenesis. By combining histological staining and transcriptomic analysis, we identified candidate genes associated with differences in ZR structure. The results suggested that stage Ⅲ of oogenesis in *C. alburnus* is a critical period for the formation of structural differences in the ZR between oocytes with contrasting spawning modes. Peptidase inhibitors, such as the *wfdc* and *a2ml* gene family, may participate in ZR structure differentiation.

## 2. Materials and Methods

### 2.1. Sample Collection

In the reproductive period (June and July), fishes of Cala and Calb of about three to four years in age (body length greater than 60 cm and body weight of 1.5–2.5 kg) were obtained from fishery facilities in Ezhou and Jinzhou, respectively, Hubei Province. The fishes were anesthetized with MS-222 and sacrificed by decapitation. The mature ovaries were placed in a petri dish and immersed in Gey’s balanced salt solution (GBSS) for follicle separation, with the processing time for each batch of ovarian samples no more than 10 min. Following Lubzens’s [18] (Lubzens et al. 2010) embryonic stage scheme, follicles at stage Ⅱ (previtellogenesis stage), stage Ⅲ (vitellogenesis stage), and stage Ⅳ (germinal vesicle migration stage) were isolated using precision tweezers. Mature eggs (stage V) were collected after inducing spawning in females via the maturation-inducing steroid (MIS) 17α, 20β-dihydroxy-4-pregnen-3-one injection. The follicles and mature eggs collected at various stages during oogenesis from Cala were designated as Cala Ⅱ, Cala Ⅲ, Cala Ⅳ, and Cala Ⅴ. Similarly, samples collected at various stages from Calb were designated as Calb Ⅱ, Calb Ⅲ, Calb Ⅳ, and Calb Ⅴ. For each developmental stage, three sample sets were collected, and each set contained three biological replicates. The follicles and eggs were first washed in 1 X phosphate buffer solution and then divided into two groups. One portion of the samples was fixed in paraformaldehyde fixative (neutral) for histological analysis and phosphate-buffered 2.5% glutaraldehyde for electron microscopy. The remaining follicles were rapidly frozen in liquid nitrogen and then stored at −80 °C for total RNA extraction. In total, eighteen Cala females and eighteen Calb females were used for the follicle and mature egg collection, respectively. For each developmental stage, three independent sample sets were collected, and each set contained three biological replicates. The follicle isolation and washing process need to be completed as soon as possible to minimize potential alterations in their properties.

### 2.2. Histological Analysis

For the light microscopy, after paraformaldehyde fixative fixation, the follicles were dehydrated in a series of graded ethanol from 75% to 100%, cleared in xylene, and embedded in Paraffin wax for 5 μm sections. Alcian Blue–Periodic Acid–Schiff (AB-PAS) staining was conducted to distinguish the glycoproteins, in accordance with routine histological procedures. The sections were observed by slide scanners Lecia Aperio VERSA 8(Leica, Wetzlar, Germany). The thickness of the zona radiata and zona radiata inner was estimated from the histological photographs and was calculated by measuring the distance from the top to the bottom of each piece using the Image J analysis software (ImageJ 1.54g). These two thicknesses were subtracted to obtain the thickness data of the outer zona radiata.

### 2.3. Transmission Electron Microscopy (TEM)

TEM was employed for a high-resolution structure analysis of the zona radiata. After a 2.5% glutaraldehyde buffer fixation, the follicles were post-fixed in 1% osmium tetroxide. The follicles were dehydrated in a series of graded ethanol from 50% to 100% and embedded in epoxy resin. Ultrathin sections of approximately 60–80 nm were stained with uranyl acetate and lead citrate and observed under transmission electron microscopy Tecnai G^2^ 20 TWIN (FEI, Hillsboro, OR, USA).

### 2.4. RNA Extraction and Quantitative Real-Time Polymerase Chain Reaction (qPCR)

The total RNA of eggs was extracted using TRIzol (Invitrogen, Carlsbad, CA, USA) according to the manufacturer’s instructions. RNA integrity was assessed using the RNA Nano 6000 Assay Kit of the Bioanalyzer 2100 system (Agilent Technologies, Santa Clara, CA, USA). A total amount of 1 μg of RNA per sample was used as an input material for the RNA sample preparations. The genomic DNA (gDNA) was removed from the total RNA with gDNA Eraser Buffer, and complementary DNA (cDNA) was synthesized from 1 μg of total RNA using HiScript^®^ Ⅲ RT SuperMix for qPCR (+gDNA wiper) (Vazyme Biotech, Nanjing, China). qRT-PCR was performed by using the ChamQ Universal SYBR qPCR Master Mix (Vazyme Biotech, Nanjing, China) on a Bio-Rad CFX96 Real-Time System (Bio-Rad, Hercules, CA, USA). The thermal cycle was set as follows: pre-denaturation at 95 °C for 2 min, followed by 40 cycles at 95 °C for 10 s and 60 °C for 30 s. The primer sequences are listed in Table S1. Actin was used as the housekeeping gene for normalization of the gene expression levels, and the data were analyzed using the 2^−ΔΔCT^ method, as described previously [37]. An independent samples t-test was performed using origin2022 to determine the significance of gene expression differences between the groups.

### 2.5. DEGs Identify, Trend Analysis, and Pathway Functional Enrichment

A differential expression analysis of different developmental stages was performed with the DESeq2 R package (1.20.0), using previously performed transcriptome sequencing, assembly, and annotation data deposited in NCBI (PRJNA932951). Genes with |log2 fold-change| (|log_2_FC|) larger than 1 and an adjusted *p*-value less than 0.05 found by DESeq2 were assigned as differentially expressed. Gene Ontology (GO) and Kyoto Encyclopedia of Genes and Genomes (KEGG) pathway enrichment analyses of differentially expressed genes were implemented by the cluster Profiler R package, in which gene length bias was corrected. The MFUZZ analysis was used to perform a time-series analysis of the DEGs during oogenesis using the “Mfuzz” R package through an online website (https://magic.novogene.com/, accessed on 22 February 2025).

## 3. Results

### 3.1. Histology and Thickness of Zona Radiata in Oocytes at Different Developmental Stages

**Figure 1:**
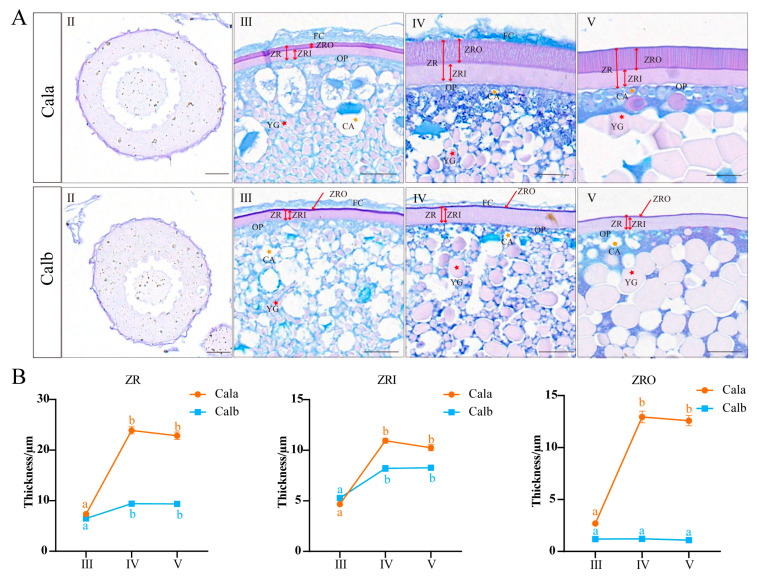
AB-PAS staining and zona radiata thickness of Cala and Calb follicles at different stages. (**A**): AB-PAS staining of follicles in Cala and Calb at different developmental stages. FC: follicle cells; ZR: zona radiata; OP: oocyte plasma; CA: cortical alveoli; YG: yolk granules; ZRI: zona radiata inner; ZRO: zona radiata outer; Scale bar: 20 μm. (**B**): Zona radiata thickness of Cala and Calb oocytes at different stages. The values are presented as the mean ± standard error (*n* = 20 per group). The different lowercase letters indicate significant differences between the three groups (*p* < 0.05, one-way analysis of variance). Ⅱ, Ⅲ, Ⅳ, and Ⅴ represent the oocytes in the picture at stages Ⅱ, Ⅲ, Ⅳ, and Ⅴ of oogenesis, respectively.

AB-PAS staining was performed on follicles across different developmental stages in Cala and Calb, respectively. In stage Ⅱ, the oocytes predominantly displayed cytoplasmic growth, with an increase in cytoplasmic proportion and nucleolar count within the oocyte. Stage Ⅲ oocytes were actively involved in vitellogenesis and exhibited a continuous and uniform ZR structure. Additionally, cortical granules began to emerge and were initially dispersed throughout the oocyte, gradually relocating closer to the oocyte plasma membrane. In stage Ⅳ, the oocytes were fully grown, with a prominent feature that the germinal vesicle began to migrate toward the animal pole, the ZR was fully developed, and the yolk granules almost filled the outer nuclear space. Oocytes in stage Ⅴ ovulated from the follicular cells, and the yolk granules became bulky as they fused to form clumps during maturation (Figure 1A). In stage Ⅲ to stage Ⅴ follicles or mature eggs, the ZR was stained purplish-red, indicating a high content of neutral carbohydrates. In contrast, the follicular cells and cortical region were stained blue, suggesting an abundance of acidic mucopolysaccharides (Figure 1A). Zona radiata thickening was primarily observed from stage Ⅲ to Ⅳ, with no significant change in thickness from stage Ⅳ to Ⅴ. The ZRO of Cala significantly thickened from stage Ⅲ to Ⅳ, whereas no such change was observed in Calb (Figure 1B).

### 3.2. Ultrastructure of Zona Radiata in Oocytes at Different Developmental Stages

**Figure 2:**
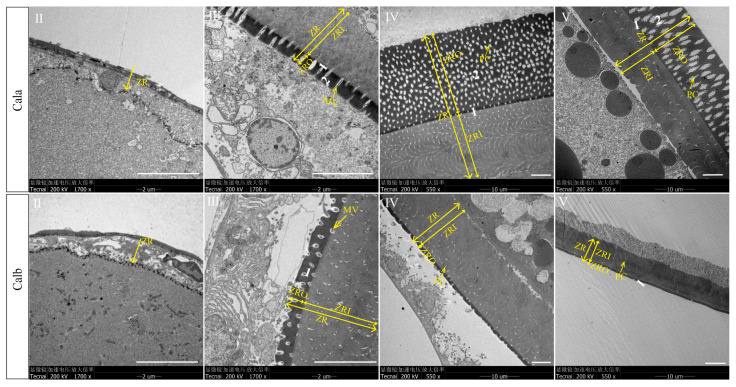
TEM of zona radiata in Cala and Calb oocytes at different developmental stages. Ⅱ, Ⅲ, Ⅳ, and Ⅴ represent the follicles in the picture at stages Ⅱ, Ⅲ, Ⅳ, and Ⅴ of oogenesis, respectively. ZR: zona radiata; ZRI: zona radiata inner; ZRO: zona radiata outer; MV: microvilli; PC: pore channel. 1 and 2 represent the layer number of ZRO. Scale bar: 5 μm. White text in the black bar shows the setting parameters of each picture: TEM brand, accelerating voltage, and magnification range. The Chinese terms in the image denote microscope, accelerating voltage, and magnification factor (from left to right).

Under higher resolution transmission electron microscopy, oocytes at stage Ⅱ were observed to begin accumulating materials to form ZR, which exhibited a discontinuous structure, and there were microvilli extending from the base of the ZR to the follicular cells. The ZR of stage Ⅲ oocytes could be divided into outer (ZRO) and inner radiation zones (ZRI) according to their electron density, and this structure is maintained until the oocytes ovulate (Figure 2). The TEM results showed that the ZRO of Cala could be divided into two layers, while the ZRO of Calb was only one layer and much thinner than that of Cala (Figure 2).

### 3.3. Transcriptome Assembly and Differentially Expressed Gene (DEG) Profiles in Follicles at Different Developmental Stages

**Figure 3:**
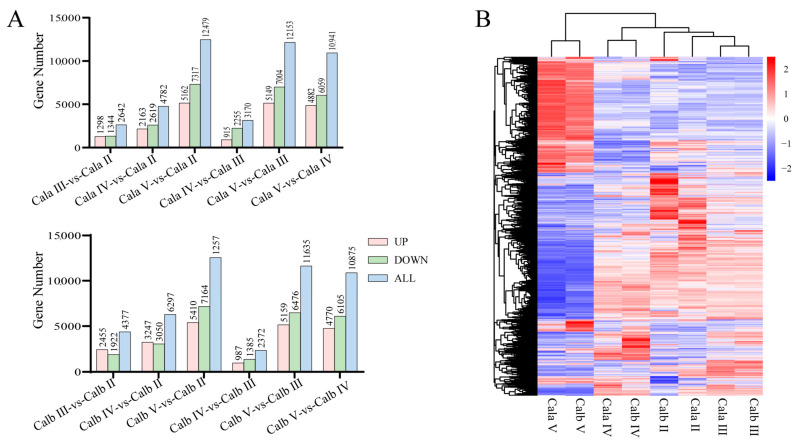
Overview of differential gene expression at each stage of oogenesis in Cala and Calb. (**A**) Number of differentially expressed genes identified by different comparison combinations in Cala and Calb. (**B**) Heatmap of the expression of 18,248 DEGs in each sample group.

DEGs in pairwise combinations at different stages of oogenesis in Cala or Calb were identified for further analysis. The clusters and numbers of upregulated and downregulated DEGs are displayed as a heatmap and a histogram, respectively (Figure 3A,B). A comprehensive analysis of the differentially expressed genes (DEGs) across six comparisons in Cala or Calb revealed a total of 15,683 and 16,386 genes that exhibited significant differential expression in one or more comparison combinations. Detailed information about the overlap of DEGs and a comprehensive list of differentially expressed genes could be found in the Supplementary Materials (Tables S2 and S3).

### 3.4. Trend Analysis of DEGs Across the Developmental Stages of Follicles in Cala and Calb

**Figure 4:**
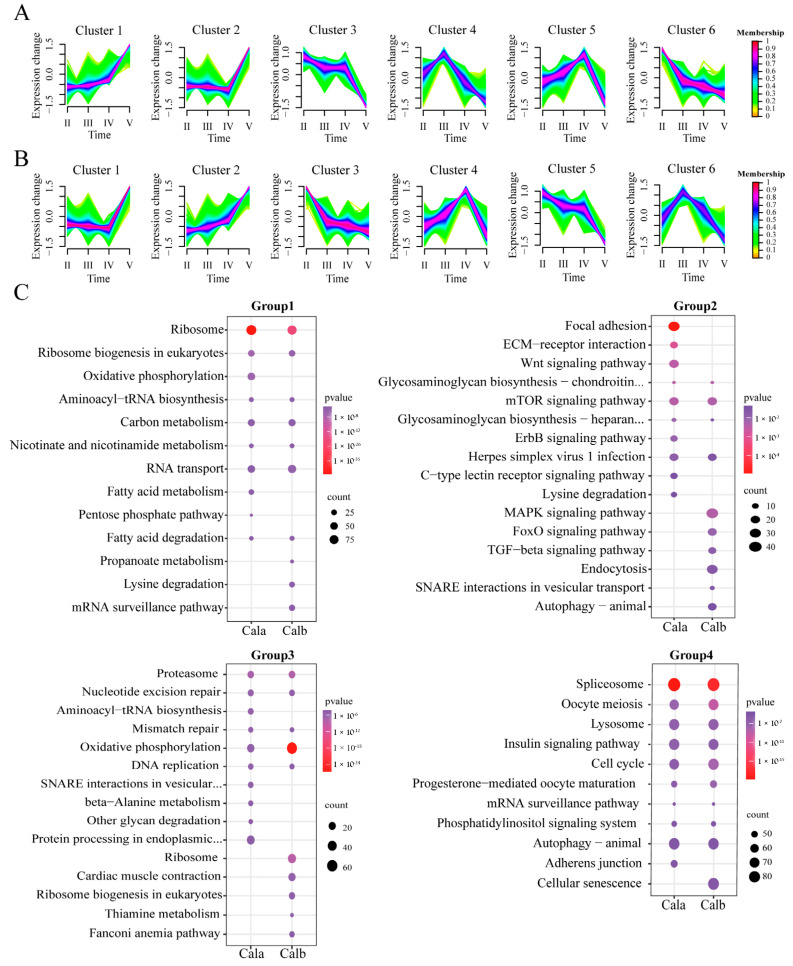
Temporal profiles of gene expression in different stage follicles from Cala and Calb. (**A**) Mfuzz clustering identified 6 distinct temporal patterns of gene expression in Cala. (**B**) Mfuzz clustering identified 6 distinct temporal patterns of gene expression in Calb. (**C**) Top10 KEGG enrichment pathways of 4 groups in Cala and Calb.

Using an Mfuzz analysis of the 15,683 DEGs in Cala and 16,386 DEGs in Calb, respectively, we observed six distinct clusters of temporal patterns representing genes that were differentially regulated, indicating different expression kinetics (Figure 4A,B). According to their gene expression trends, the clusters with the highest expression levels in the same period were classified into one group, and the four groups had the highest expression levels in stage Ⅱ (cluster 3 and cluster 6 in Cala, cluster 3 and cluster 5 in Calb), Ⅲ (cluster 4 in Cala, cluster 6 in Calb), Ⅳ (cluster 5 in Cala, cluster 4 in Calb), and Ⅴ (cluster 1 and cluster 2 in Cala and Calb), respectively.

Group 1 genes in Cala and Calb, which exhibited the highest expression at stage Ⅱ, were both significantly enriched in protein synthesis and molecular metabolism-related pathways, such as ribosome (dre03010, *p*-value < 0.001), ribosome biogenesis in eukaryotes (dre03008, *p*-value < 0.001), carbon metabolism (dre01200, *p*-value < 0.001), and fatty acid degradation (dre00071, *p*-value < 0.05) (Figure 4C). The enrichment results of group 2 genes in Cala included some pathways related to the synthesis of extracellular matrix molecules that were not found in Calb, such as focal adhesion (dre04510, *p*-value < 0.001) and ECM-receptor interaction (dre04512, *p*-value < 0.01), while glycosaminoglycan biosynthesis pathways—chondroitin sulfate (dre00532, *p*-value < 0.01) and heparan sulfate (dre00534, *p*-value < 0.05)—were both found in the enrichment of group 2 genes for Cala and Calb (Figure 4C). The pathways associated with DNA structure enriched from group 3 genes in both Cala and Calb were as follows: mismatch repair (dre03430, *p*-value < 0.05) and nucleotide excision repair (dre03420, *p*-value < 0.001) (Figure 4C). Group 4 genes were enriched in several oocyte maturation and meiosis pathways: oocyte meiosis (dre04114, *p*-value < 0.001), progesterone-mediated oocyte maturation (dre04914, *p*-value < 0.001), and cell cycle (dre04110, *p*-value < 0.001), which were highly overlapped in both Cala and Calb (Figure 4C).

### 3.5. Genes Related to the Zona Pellucida (ZP) Proteins and Glycosaminoglycan Biosynthesis

**Figure 5:**
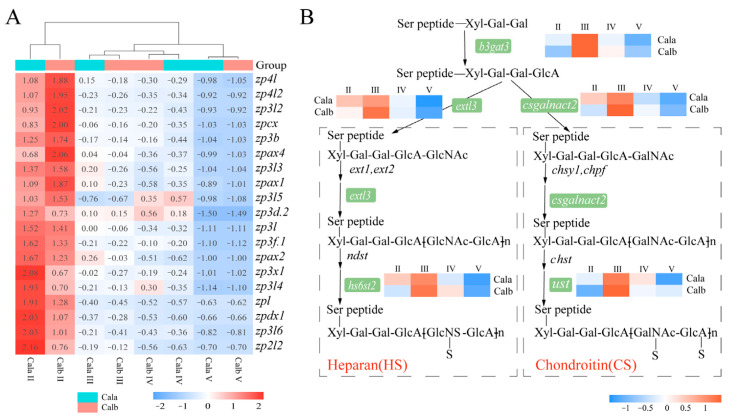
Heat map of *zp* and glycosaminoglycan biosynthesis-related genes. (**A**) Heat map of *zp* gene expression levels in Cala and Calb follicles at each stage. (**B**) Simplified model of the heparan and chondroitin biosynthesis pathway.

More than 20 *zp* genes were identified with the transcriptome data. In follicles from both Cala and Calb, most of the *zp* genes showed a decreased expression pattern from stage Ⅱ to stage Ⅴ (Figure 5A). Among these *zp* genes, *zp2l2* and *zp3x1* showed the largest fold-change in expression between Cala and Calb during stage II (Table S4). Additionally, several genes related to glycosaminoglycan biosynthesis were clustered to group 2 through Mfuzz and KEGG analyses, such as *b3gat3*, *extl3*, *csgalnact2*, *hs6st2*, and *ust*. These genes were most highly expressed at stage Ⅲ during oogenesis and involved in the synthesis of the protein linkage region, chain elongation, and sulfated structures of sulfated glycosaminoglycans, such as heparan sulfate (HS) or chondroitin sulfate (CS) (Figure 5B).

### 3.6. Comparative Transcriptome Analysis of Cala and Calb Follicles at Stage Ⅲ

**Figure 6:**
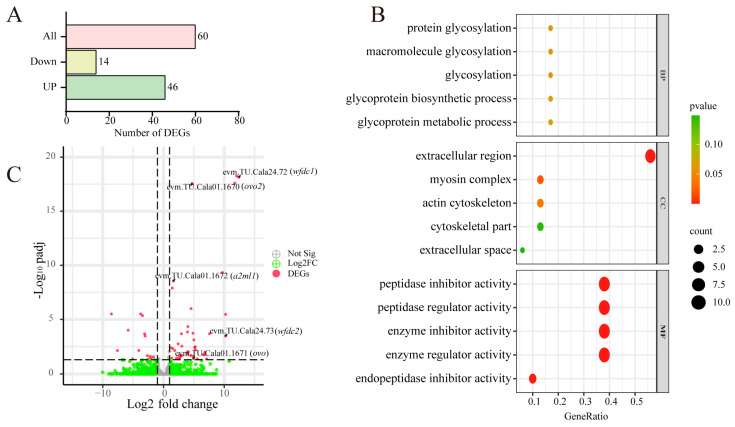
Transcriptome analysis of differences between Cala and Calb. (**A**) Number of DEGs identified in a transcriptome comparison of stage Ⅲ follicles in Cala and Calb. (**B**) GO enrichment of the 46 upregulated genes. (**C**) Volcano plot of differentially expressed genes in the comparative transcriptome of stage Ⅲ follicles in Cala and Calb.

To pinpoint candidate genes involved in regulating ZR structural differences, we performed a comparative analysis of the transcriptional profiles between follicles at stage Ⅲ from Cala and Calb. The comparative transcriptomic analysis identified a total of 60 differentially expressed genes, of which 46 genes were upregulated in Cala (Figure 6A). GO enrichment analyses of these upregulated genes showed that their functions were mainly enriched in glycoprotein synthesis, extracellular region and cytoskeleton synthesis, and peptidase activity regulation (Figure 6B). The genes that are upregulated mainly include the WAP-type (Whey Acidic Protein) ‘four-disulfide core’ (*wfdc*) gene family, alpha-2-macroglobulin-like (*a2ml*) family, collagen, and myosin (Figure 6C, Table 1).

### 3.7. qRT-PCR to Verify DEGs

**Figure 7:**
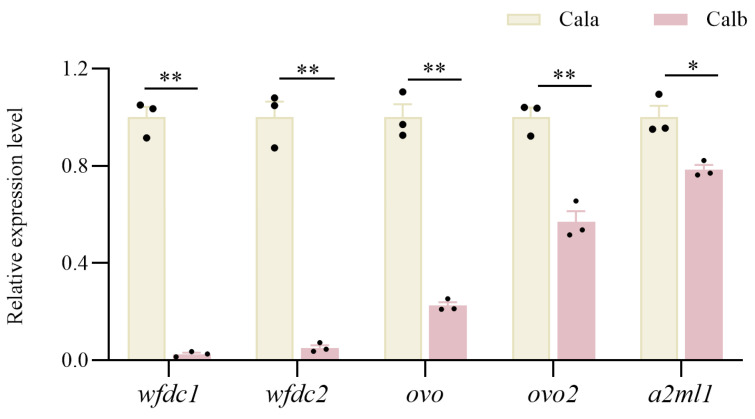
qRT-PCR results of key DEGs. The qPCR analyses of genes associated with a peptidase inhibitor in Cala and Calb follicles at stage Ⅲ. Gene expression levels are shown relative to those of Calb follicles. Each bar represents the mean ± standard error (SE) (*n* = 3). * indicates *p* < 0.05, and ** indicates *p* < 0.01.

qPCR was carried out to verify the expression variations of five peptidase activity regulation-related genes, which were upregulated in Cala screened from the Cala and Calb stage Ⅲ follicle comparative transcriptome. The results showed that the expression differences of these genes, detected by qPCR, were consistent with the changes determined by the RNA-Seq expression analysis, which were all significantly upregulated, suggesting that the results of the RNA-Seq expression analysis are reliable. (Figure 7).

## 4. Discussion

### 4.1. Structure Formation Process and Temporal Gene Expression Patterns Associated with ZR Development Across Different Oogenesis Stages

The teleost oocyte is enveloped by a protective non-cellular membrane known as ZR, protecting the eggs from adverse physical and chemical environments while facilitating fertilization and subsequent development [12]. Initial formation of the ZR was suggested to be at the late perionucleolar stage [38] or earliest cortical alveoli stage [39], with both stages occurring before the onset of vitellogenesis [39,40]. In *C. alburnus*, TEM images revealed that ZR formation initiated at stage Ⅱ, which corresponded to the previtellogenic stage, and progressively thickened through stage Ⅲ and stage Ⅳ. ZR formation starts at the base of the microvilli and extends outward through material deposition and glycoprotein organization. When the cell developed to stage Ⅳ, the microvilli retracted, leaving empty pores within the ZR. Proper retraction of these microvilli is required for the separation of the mature egg and the follicular cell layer, thus preparing the oocyte for ovulation [18,41]. In general, the microvilli structure changes are conserved across teleosts and likely contribute to connecting the oocyte to the follicular cell layer as well as facilitating ZR formation [18,20,22,41].

The ZR in fish oocytes is primarily composed of zona pellucida (ZP) proteins, which are glycosylated proteins containing a single ZP domain [10,42]. ZP deposition in the ZR process includes precursor synthesis, glycosylation modification, vesicle transport, and, finally, assembly within the ZR [43,44]. Similarly to findings in Japanese eel (*Anguilla japonica*) [32,33], the expression of most *zp* genes in Cala was also highest at stage Ⅱ. Integrating insights from TEM imaging and gene expression analysis highlights that stage Ⅲ of oogenesis is critical for ZRO formation and maturation.

### 4.2. Structural Differences in ZR Between Cala and Calb

The structural variation between the ZR of Cala and Calb oocytes during oogenesis is most evident in its thickness and the number of layers. The differences in ZR structure between Cala and Calb were initially observed when the oocytes developed to stage Ⅲ through histological staining and TEM. ZRO in Cala was thicker and could be further classified into two layers based on variations in electron density, while ZRO in Calb was thinner and exhibited a uniform single-layer structure. ZR structure and thickness are species-specific and influenced by habitat and spawning grounds [45,46]. The ZR of adhesive eggs tends to be thicker than those in semi-buoyant eggs, which reflects the different ability to resist mechanical stress [1]. The observed structural divergence between Cala and Calb is consistent with other Cyprinid species endemic to East Asia, where similar variations in ZR thickness and layering are noted between adhesive and semi-buoyant eggs [1]. Similarly to the case reported in aplochelioid killifishes, the characteristics of ZR can better reflect the convergence of ecological adaptation among species than their genetic relationship [47,48]. These observations suggest that the thickness and layering of the ZR are key factors in eggs’ adaptation to its respective reproductive strategy, where adhesion to a substrate requires a more fortified envelope.

### 4.3. Molecular Mechanisms Underlying Differences in ZR Structure

Differences in ZR structure between Cala and Calb are likely driven by distinct molecular pathways that are related to ZP components, GAG biosynthesis, and protease activity regulating ZR stability and thickness.

The structures of the various layers of ZR are composed of different ZP proteins [10,26]. The ZR of Cala exhibited significantly higher abundance of ZP2 and ZP3X1 proteins compared to the Calb, with ZP3X1 showing specific localization within the ZRO exclusively in Cala [1]. Transgenic zebrafish lines expressing the *zp3a* gene from rare minnow (*Gobiocypris rarus*) produced fertilized eggs exhibiting partial adhesiveness, with the formation of protrusion structures on the ZR resembling those of *G. rarus* [49]. Distinct compositional profiles of ZP proteins may constitute the structural basis for ZR structure differences [10,25]; however, the regulatory mechanisms driving their differential expression within follicular microenvironments require further investigation.

In addition to differences in ZP protein composition and abundance, eggs of Cala develop an additional outer adhesive layer enriched in acidic polysaccharides after fertilization, which is absent in the Calb eggs [1]. Similarly, in fertilized carp eggs, glycosidase treatment does not affect egg adhesiveness, whereas protease treatment abolishes adhesion and removes the PAS-positive outer envelope layer, indicating that adhesive properties primarily rely on the proteinaceous components, particularly glycoproteins [11]. The formation of egg adhesiveness is mediated by materials diffusing from the cortical alveoli within 30s of fertilization [27,50]. A comparative transcriptomic analysis of follicles at different developmental stages in *C. alburnus* revealed that several genes involved in GAG biosynthesis, including *b3gat3*, *extl3*, *hs6st2*, *ust*, and *csgalnact2*, exhibited peak expression during stage Ⅲ of oogenesis. *B3gat3* encodes β-1,3-glucuronyltransferase I (GlcAT-I), which catalyzes the initiation step of GAG biosynthesis, a shared process in both heparan sulfate and chondroitin sulfate synthesis [51,52]. The polymerization of HS chains relies on the coordinated catalytic activity of glycosyltransferases EXT1, EXT2, and EXTL3, which sequentially add disaccharide units to elongate the chain. Subsequent modifications are mediated by HS-specific sulfotransferases, such as HS3ST1 and HS6ST2, which transfer sulfate groups to glucosamine residues and generate characteristic sulfation patterns [51]. In the CS biosynthetic pathway, *csgalnact2* encodes chondroitin sulfate N-acetylgalactosaminyltransferase 2 (CSGALNACT2), responsible for chain elongation, while *ust* encodes uronyl 2-O-sulfotransferase (UST), which catalyzes the 2-O-sulfation of glucuronic acid residues in the CS chains [53,54]. Moreover, *extl3* and *ust* exhibit significantly higher expression levels in the mature ovaries of Cala compared to Calb [1]. The extensive formation of cortical alveoli and the peak expression of genes involved in the glycosaminoglycan biosynthesis during stage Ⅲ of oogenesis suggest that gene activity at this stage may play a critical role in the synthesis of glycosaminoglycan inclusions within cortical alveoli and even the formation of the adhesive layer.

Protease and protease inhibitor activities play a key role in maintaining the structure and thickness of ZR [55,56]. Prss59.1, a trypsin-like proteolytic enzyme, is involved in chorion elevation and in the proper formation of the ZR in zebrafish [56]. Fertilized eggs of *Tribolodon hakonensis* treated with PCMB or L-cysteine, which inactivate the chorion protease inhibitor WFDC protein, exhibit fragile ZR and reduced resistance to physical environmental stress [55,57]. Furthermore, the overexpression of the mouse *wfdc* gene in HC11 cells inhibits the pancreatic elastase-mediated cleavage of laminin and enhances cell adhesion [58]. *A2ml1*, identified as a promising candidate gene for ZR structure regulation through a transcriptomic analysis of follicles from Cala and Calb, has been reported to inhibit MMPs activity, thereby contributing to the structural remodeling of the extracellular matrix [59,60]. Comparative proteomic analyses of ZR conducted one hour post fertilization revealed significantly higher levels of A2ML1 protein in the ZR of common carp adhesive eggs compared to grass carp semi-buoyant eggs [13]. The significantly upregulated expression levels of *wfdc* and *a2ml* in Cala follicles suggested that these genes may play a role in regulating protein deposition in the ZR, thereby contributing to the differences in ZR thickness between adhesive and semi-buoyant eggs.

## 5. Conclusions

In this study, we integrated histological staining, transmission electron microscopy, and comparative transcriptomics to analyze the temporal formation of structural differences in the zona radiata between different spawning habits of *C. alburnus*, as well as their underlying molecular mechanisms. Histological staining revealed that the structural variances in the zona radiata of adhesive and semi-buoyant eggs initiated during stage Ⅲ of oogenesis. Through comparative transcriptomics of stage Ⅲ follicles, we inferred that elevated expression levels of GAG synthesis and peptidase inhibitors potentially contribute to the increased thickness of the zona radiata in adhesive eggs. These findings provide a theoretical basis for understanding the molecular mechanisms underlying the divergence in spawning habits among teleosts.

## Figures and Tables

**Table 1:** Differentially expressed genes associated with the egg envelope structure of Cala and Calb at stage Ⅲ.

Gene ID	Description	Log_2_ (Fold Change ^1^)	GO Enrichment
novel. 2876	WAP-type (Whey Acidic Protein) ‘four-disulfide core’	15.04 ***	extracellular region/peptidase inhibitor
novel. 868		15.09 ***	
novel. 3103		13.10 ***	
evm. TU. Cala24.72		12.42 ***	
novel. 3101		11.75 ***	
novel. 3499		9.70 ***	
novel. 742		10.22 ***	
evm. TU. Cala24.73		10.22 ***	
evm. TU.Cala01.1672	Alpha-2-macroglobulin-like protein 1	1.62 ***	
evm. TU.Cala01.1670	Ovostatin homolog 2	4.75 ***	peptidase inhibitor
evm. TU.Cala01.1671	Ovostatin	4.91 *	peptidase inhibitor
evm. TU.Cala14.543	Collagen alpha-1(XIV) chain	2.14 *	cytoskeleton
evm. TU.Cala24.648	Myosin-7B	7.10 *	cytoskeleton

^1^ The fold changes are indicated as compared with the stage Ⅲ follicles of Calb. Genes with a fold difference > 2 were considered significantly altered, which are indicated with * (*p* < 0.05) or *** (*p* < 0.001).

## Data Availability

The raw transcriptomic datasets generated and analyzed during the current study are available from the corresponding author upon request.

## Supplementary Materials

The following supporting information can be downloaded at: https://www.mdpi.com/article/10.3390/biology14080987/s1, Table S1: primer sequence used for Q-PCR; Table S2: gene ID of DEGs identified in Cala; Table S3: gene ID of DEGs identified in Calb; Table S4: FPKM of *zp* genes in Cala follicles across different developmental stages.

## Abbreviations

The following abbreviations are used in this manuscript:

*wfdc*	WAP-type (Whey Acidic Protein) ‘four-disulfide core’
*a2ml*	alpha-2-macroglobulin-like
ZR	zona radiata
GAG	glycosaminoglycan
ZGA	zygotic genome activation
Cala	*C. alburnus* producing adhesive eggs
Calb	*C. alburnus* producing semi-buoyant eggs
ZP	zona pellucida
FPKM	Fragments Per Kilobase of transcript per Million mapped reads
Mfuzz	a fuzzy c-means clustering algorithm
